# Anticancer potential of Thymoquinone from *Nigella sativa* L.: An in-silico and cytotoxicity study

**DOI:** 10.1371/journal.pone.0323804

**Published:** 2025-06-12

**Authors:** Yathendranaik Ravi, Periyanadar Irene Vethamoni, Shailendra Nath Saxena, Muthusamy Kaviyapriya, Vichangal Pridiuldi Santhanakrishnan, Muthurajan Raveendran, Narayana Naik Ashoka, Sharda Choudhary, Arvind Kumar Verma, Chowdasandra Byregowda Harisha, Palanisamy Dhamotharan, Vinay Bhardwaj

**Affiliations:** 1 HC & RI, Tamil Nadu Agricultural University, Coimbatore, Tamil Nadu, India; 2 ICAR-National Research Centre on Seed Spices, Ajmer, Rajasthan, India; 3 Centre for Plant Molecular Biology and Biotechnology, Tamil Nadu Agricultural University, Coimbatore, Tamil Nadu, India; 4 College of Horticulture, Sirsi, University of Horticultural Sciences, Bagalkot, Karnataka, India; 5 ICAR-National Institute of Abiotic Stress Management, Pune, Maharashtra, India; 6 Centre for Genetics and Plant Breeding, Tamil Nadu Agricultural University, Coimbatore, Tamil Nadu, India; Shiraz University of Medical Sciences, IRAN, ISLAMIC REPUBLIC OF

## Abstract

*Nigella sativa* L. widely used spice cum medicinal plant in Asia and the middle east, is renowned for its seeds and oil which possess both culinary and therapeutic purposes. Its rich content of bioactive compounds, including metabolites and phenolics, with Thymoquinone, a monoterpene quinone, emerging as key therapeutic compound significantly consideration for its various pharmacological activity with lower toxicity compared to conventional chemotherapy. This study evaluated the anticancer potential of thymoquinone isolated from *N. sativa* L., through cytotoxicity and *In Silico* studies. Seeds from 38 accessions were collected across the country and screened for Thymoquinone content using HPTLC with the highest concentration identified in Ajmer Nigella 13 (247.60mg 100gm^−1^) accession. *In Vitro* MTT assay of Thymoquinone in human myelogenous leukemia (K562) cells demonstrated significant dose and time dependent cytotoxicity confirming Thymoquinone’s potential as a promising therapeutic candidate for leukemia and other cancer.

## 1. Introduction

Black seed or *kalonji* (*Nigella sativa* L.) is a spice cum medicinal plant from the Ranunculaceae family [1] and it possesses polyphenols, flavonoids and other bioactive metabolites [2]. The seeds comprise of 15–30% fixed oil, primarily composed of non-saturated acids *viz.,* oleic, linoleic and palmitic acids. It also includes 11,14-cis, cis-eicosadienoic acid [3]. It is a promising medicinal plant and holds a wide range of pharmacological activities, including antibacterial [4], antioxidant [5], anti-inflammatory [6], anti-diabetic [7], anticancer [8] and anti-nausea [9] activities. The primary constituents of the seed’s volatile oil (0.5–1.6%) are monoterpenes α-pinene, p-cymene, β-pinene, α-thujene, γ-terpinene, carvacrol and thymoquinone [10]. Phenolic compounds have been demonstrated to exhibit an array of biological actions, including anti-cancer properties. Studies have demonstrated that these compounds can inhibit the cancer cells growth, induce apoptosis and prevent angiogenesis that supplies tumors with nutrients [11]. Beyond their anti-cancer properties, phenolic compounds also possess anti-inflammatory, potentially reducing the risk of chronic diseases [12]. These substances include an aromatic ring containing one or additional hydroxyl groups that contribute an array of functions, including antimicrobial activity, radical scavenging activity and defensive mechanisms in plants [13].

Cancer is a devastating disease that affecting millions globally, leading to high morbidity and mortality rates. The search for effective anticancer therapies has been ongoing for many years and one promising avenue of research involves the use of natural compounds derived from medicinal plants [14]. Combating resistance to targeted cancer therapies involves combination strategies, using multiple drugs that target various molecular pathway intricate in cancer cell growth, survival and increase. Advances in identifying new therapeutic targets have improved patient’s survival rates by reducing chemotherapy, delaying treatment, late diagnosis and cancer recurrence [15].

Thymoquinone chemically 2-isopropyl-5 methyl-1,4-benzoquinone is a key active metabolite in the seed oil of *Nigella sativa* L. The anticancer potential of Thymoquinone  has been demonstrated through the downregulates MUC4 via the proteasomal pathway, induces apoptosis and reduces motility and migration in pancreatic cancer cells [16]. Activation of PPAR-β/δ and PPAR-γ, which reduced apoptosis upon PPAR-γ inhibition [17], inhibits STAT3 and reduces survival proteins in gastric cancer and inhibits oral cancer by downregulating p38β MAPK [18], interacts with PAK1, disrupts MEK-ERK1/2 signaling and enhances apoptosis in colorectal cancer [19], inhibits NF-κB, reduces IL-8 expression, induces G2M arrest, increases ROS and promotes apoptosis in Hepatocellular carcinoma [20], exhibits strong anticancer effects in breast cancer by inducing apoptosis and cell cycle arrest, thereby reducing proliferation and migration in MCF7 and MDA-MB-231 cells [21], modulating signaling pathways, regulating cell cycle proteins, inducing apoptosis and upregulating growth factors like PTEN and VEGF, making it a promising target for treating aggressive breast cancer types, including Triple-negative Breast Cancer [22–24] and nanomedicine formulations enhance thymoquinone ability to cause DNA damage and inhibits tumor progression by activating caspases and suppressing anti-apoptotic proteins in breast cancer cells [25]. Similarly, various anti-cancer activities induce reactive oxygen species, aneuploidy and apoptosis, with resistance linked to NQO1 and GSH activity in carcinoma cell [26], regulate oncogenic signalling [27], induces apoptosis, inhibits metastasis, improves immune response in osteosarcoma and bone metastasis [28], suppresses colorectal cancer by modulating key cellular processes [29].

Studies have shown Thymoquinone  exhibits anti-inflammatory properties by downregulating cyclooxygenase-2 (COX-2) levels, reducing prostaglandin production and mitigating inflammatory responses in both airway and immune cells (5), alleviates anti-inflammatory and immunomodulatory effects through modulating IL-6 signalling and reducing inflammation [30]. Also modulates inflammatory mediators and protects against autoimmune diseases by regulating cytokine production [31,32], enhancing regulatory T cell activity [33], controlling arachidonic acid metabolism, regulating signalling pathways and reducing oxidative stress [34,35]. In addition, Thymoquinone improves folliculogenesis, increases FSH and GPx1 and reduces atretic follicles, ovarian cysts, luteinizing hormone and testosterone in polycystic ovary syndrome [36].

AMPK activation by thymoquinone was shown to reduce fatty acid synthesis, enhance oxidation, promote white adipose tissue browning to increase thermogenesis and reprogram lipid and sterol synthesis [37], while the downregulation of PPARγ expression led to the activation of genes involved in adipocyte differentiation through the PPARγ/C/EBPα pathway, suggesting its potential anti-obesity properties [38], improved obesity by reducing body weight, fat formation and adipocyte hypertrophy while normalizing fat metabolism through AMPK regulation [39] and alleviated diet-induced obesity and hyperglycemia by regulating the expression of SREBP-1c, C/EBPα, FAS and IRS-1 through the AMPK pathway, thereby improving glucose tolerance, insulin sensitivity and lipid metabolism [40].

The ability to improve lipid profile, regulate lipoprotein metabolism and modulate cholesterol synthesis and transport highlights thymoquinone’s potential in metabolic disorders such as dyslipidaemia [41]. Further, the Thymoquinone  reduced MAP, total cholesterol and LDL levels in hypertensive condition, with its antihypertensive activity mediated through angiotensin II receptor blocking rather than ACE inhibition [42]. Thymoquinone with nanoformulations enhanced their effectiveness in lowering blood pressure highlighted their therapeutic value in managing hypertension [43].

Bioactive compounds mitigate the harmful effects of chemotherapy by acting as antioxidants, anti-inflammatory agents and anti-apoptotic properties, which protect healthy cells from damage. They improve treatment outcomes by regulating cell signaling pathways and boosting immune responses. The antioxidant and anti-inflammatory properties could reduce organ damage caused by drugs like cisplatin and methotrexate [44], reduce nephrotoxicity caused by cisplatin, a chemotherapeutic drug [38], alleviate the adverse effects associated with chemotherapy treatment, protective effects on spermatogenesis and testicular health [45], reduce chemotherapy-induced testicular damage suggests preserving male fertility and reproductive health during chemotherapy [46].

The research findings of thymoquinone the primary bioactive compound of *Nigella sativa*  highlights its pharmacological properties against various diseases, reinforcing its potential as a promising therapeutic agent. Advances in *In Silico* methods have provided detailed molecular insights into the mode of action of thymoquinone with specific target receptors, facilitating the development of targeted metabolite therapies. These computational approaches enable precise prediction of how bioactive compounds interact with disease-related proteins, aiding in the identification of binding sites, molecular stability and interaction [47]. This helps to optimizes drug design by suggesting structural modifications to improve efficacy and reduce side effects but also accelerates drug discovery by reducing the need for extensive *in vitro* and *in vivo* experiments [48]. Molecular docking, in particular, has emerged as a key tool in predicting the binding affinity and specificity of thymoquinone  with cancer-related proteins, contributing to the identification of potential therapeutic targets [49].

The objective of this study was to evaluate the anticancer potential of thymoquinone isolated from *Nigella sativa* L. by assessing its cytotoxicity against the human myelogenous leukemia (K562) cell line using the MTT assay and analysing its molecular interactions with cancer targets through *In Silico* methods, in comparison to Busulfan, to determine its efficacy and potential as a less toxic alternative for cancer therapy.

## 2. Materials and methods

### 2.1. Collection of plant sample

The seeds of *N. sativa* L., from the Ajmer Nigella 13 accession were collected from the ICAR- NRC on Seed Spices, Tabiji, Ajmer. The bioactive compound Thymoquinone extracted to assess its anticancer activity via cell line [human myelogenous leukemia (K562)] studies.

### 2.2. Extraction of Thymoquinone from N. sativa L. seeds

For the extraction of oil from seeds of Ajmer Nigella 13 accession a Soxhlet apparatus wherein powdered dry seed (15g) was immersed in 50 ml of absolute *n-*hexane at 65°C for 6 hours with minor modifications and the recovered extract was subjected to rotary flash evaporation under low pressure to get rid of residual solvent, then the odours oil was collected based on the AOAC procedures [50]. HPLC analysis was done using an Agilent 1200 HPLC system with a diode array detector (Agilent Technologies, Palo Alto, USA) (HPLC-DAD). A C18 column (250 x 4.6 mm ID, 5 µm particle size, Agilent Technologies, USA) was being used in a water: methanol (40:60 proportion) mobile phase that was sonicated at 37°C for 10 minutes and filtered using a 0.45 mm Millipore filter, and the injection volume was 20 *μ*L. Thymoquinone was identified at a wavelength of 254 nm under ambient temperature. A 1.5 mL/min flow rate was utilised, and identification was validated by comparing the time.

**Fig 1:**
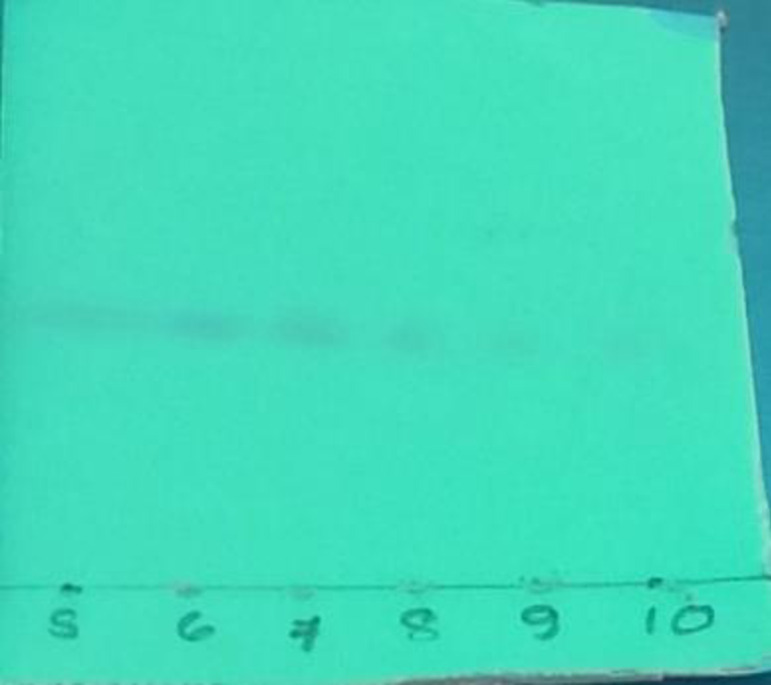
TLC Plate Showing Standard Thymoquinone (S) and Purified Fractions.

Thin Layer Chromatography (TLC) plates (Merck, Darmstadt, Germany) with the specifications of silica gel 60G, with dimensions of (4.5 mm x 10 mm), was used to identify thymoquinone. The development system was made up of *n*-hexan: ethyl acetate (8:2 v/v) produced a strong and well-defined band for the metabolite and the identity was validated by comparing the bands of standard Thymoquinone with those of studied sample extracts, as well as the *Rf* (0.56) of the reference with that of the sample [51] (Fig 1).

### 2.3. Purification of Thymoquinone by column chromatography

After extraction of the total oil, the oil slurry was made by combining it with a 1:2 ratio of silica gel (silica gel made of Sigma Aldrich, high purity grade, 60741, pore size: 60Aº, 70–230 mesh, 6–200µm). The slurry was then put through column chromatography. Hexane was used to load the column and the fractions were obtained by progressively increasing the polarity of the hexane solution up to 10% analytical grade ethyl acetate. The existence of Thymoquinone in the deployed fraction was verified by thin layer chromatography (TLC) using a 30% ethyl acetate in a hexane solution. Following the collection of pure Thymoquinone, the fractions collected in the tube numbers 6–29 were combined and HPLC was used to verify the purity once more.

### 2.4. In-vitro anticancer assay

The isolated metabolite from *N. sativa* L., seed samples was tested in vitro for cytotoxicity using the MTT assay in human myelogenous leukaemia (K562) cell lines (supplied from the National Centre for Cell Science in Pune). The assay was carried out for the samples in accordance with ISO 10993:5 criteria. In aseptic conditions, the cells were seeded in 96-well microtiter plates and cultured at 37°C with a 5% CO2 flow. Samples used for testing (varying amounts of pure Thymoquinone, i.e., 5, 25, 50, 75, 100µM ml^−1^) for 24, 48 and 72 hours in three replications were added to the cells. Following this, each well received 50 µL MTT solution (at 1 mg mL^−1^ MTT in saline solution with phosphate buffer [pH 7.2]), which was then incubated for four hours. Cell viability was evaluated before the tests in accordance with the methodology of [52].

The formula used to calculate the cytotoxicity and cell viability is as follows:


$$Cytotoxicity=\left(\frac{[Control-Sample]}{Control}\right)\times 100$$

$$Cell\;viability=\left(\frac{Sample}{Control}\right)\times 100$$

**Fig 2:**
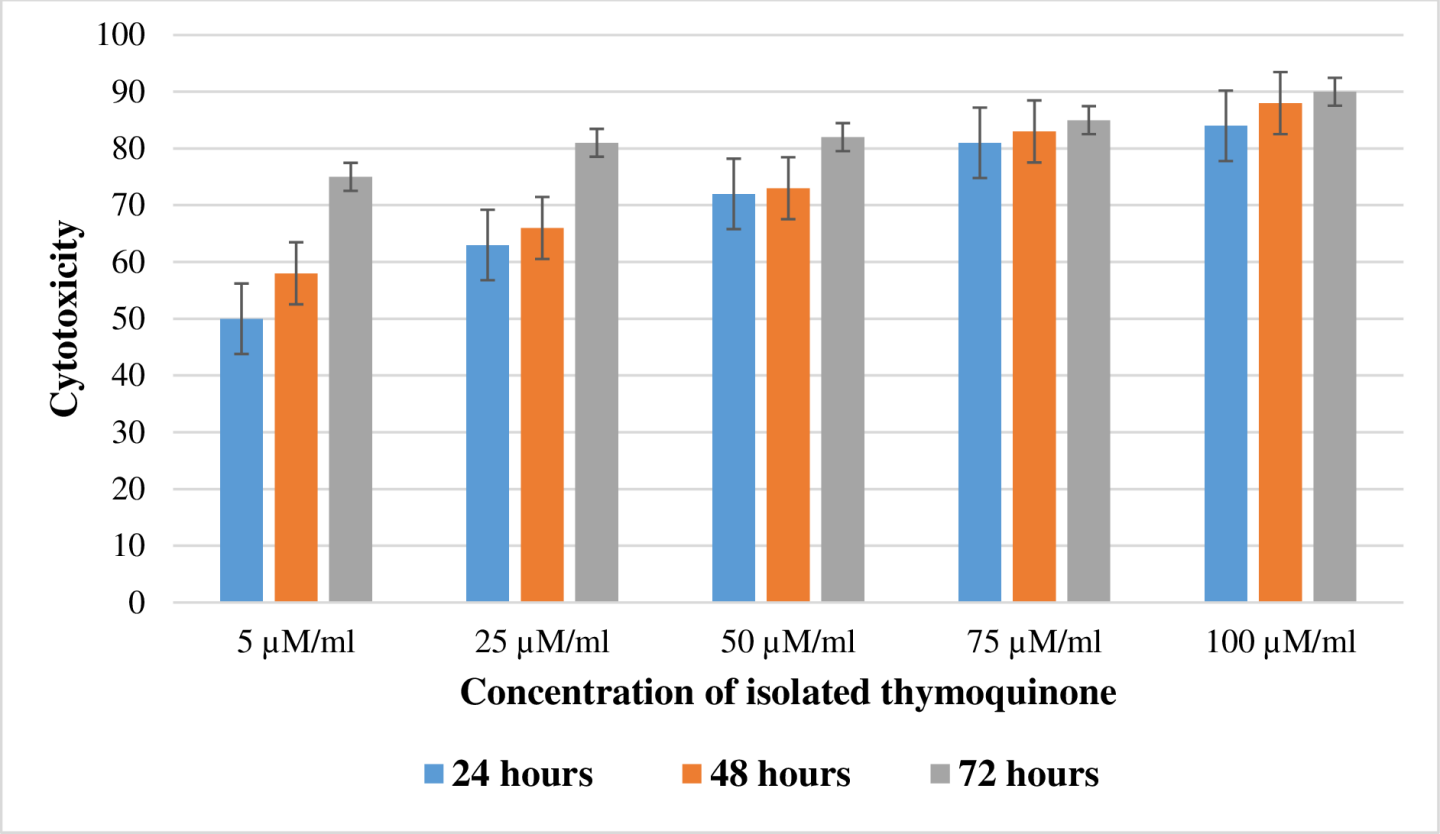
*In vitro* Cytotoxicity Study Using MTT Assay Against K562 Cell Line.

The relevant percent cell viability and cytotoxicity percentage with different concentrations and different time intervals of Thymoquinone were connived in the form of a bar graph and depicted in Fig 2.

### 2.5. Morphological investigation

The changes in the cell morphology of K562 cells subjected to concentrations ranging from 0.01–1 mg ml^−1^ of isolated Thymoquinone metabolite were measured using an inverted phase contrast microscope (OLYMPUS CKX 41) at a magnification of 20X.

### 2.6. Computational study: Using molecular docking to explore anticancer potential

#### 2.6.1. ADMET prediction of Thymoquinone and Busulfan.

The Absorption, Distribution, Metabolism, Excretion and Toxicity (ADMET) characteristics of the amalgams were evaluated to gain insights into their pharmacokinetic profiles. The canonical SMILES for the chosen compounds, Thymoquinone and Busulfan, were obtained from the PubChem database and entered into the Swiss ADME tool available on the Swiss Institute of Bioinformatics (SIB) website.

#### 2.6.2. Selection of leukemia targets.

The target proteins associated with leukemia were identified through an extensive literature review to involved in various activities in leukemia. The 3D X-ray quartz structures of these targets were sourced from RCSB Protein Data Bank and are listed in Table 1 [61]. The significance of the selected targets in leukemia is summarized in Table 1. In this study Busulfan is chosen as standard for comparing the natural compound, Thymoquinone.

**Table 1:** The selected protein targets for leukemia and their role.

S. No	leukemia targets	Role
1	MMP2	MMP2 disrupt tumor progression and metastasis, improving treatment outcomes and potentially reducing the risk of cancer spread [53].
2	AKT	AKT inhibitors disrupt cancer cell growth by blocking AKT activity, which impairs cell survival, proliferation and resistance to apoptosis. This results in reduced tumor growth and enhanced sensitivity to other therapies [54].
3	PTEN	PTEN helps maintain genomic stability by regulating the cell cycle and DNA repair. It also influences cell movement and adhesion, affecting cancer spread and metastasis. By inhibiting the PI3K/Akt pathway, PTEN controls cell proliferation and survival, thereby preventing uncontrolled cell growth and tumor development [55].
4	EGFR	EGFR (Epidermal Growth Factor Receptor) is a cell surface receptor that, when activated, triggers signaling pathways involved in cell growth, differentiation and survival. Overexpression or mutations in EGFR are commonly associated with various cancers, leading to uncontrolled cell proliferation and tumor progression [56].
5	LYN	LYN is a tyrosine kinase that regulates immune cell signaling, cell proliferation and survival. In cancer, its dysregulation can contribute to tumor growth and immune evasion. Elevated LYN activity in some leukemas and lymphomas makes it a promising drug target [57].
6	VEGFR2	VEGFR2 (Vascular Endothelial Growth Factor Receptor 2) is a receptor that mediates angiogenesis by regulating the formation of new blood vessels. Over activation or dysregulation of VEGFR2 is associated with tumor growth and metastasis by enhancing blood supply to tumors [58].
7	TGF-β3	TGF-β3 regulates cell growth, differentiation and extracellular matrix remodelling, playing a key role in wound healing and development. In cancer, it can act as both a tumor suppressor and supports angiogenesis and enables immune escape, contributing to an oncogenic environment [59].
8	CD44	Drugs targeting cancer stem-like cell markers (CD44, CD133, ALDH1) aims to eliminate cancer stem cells, reducing tumor growth, drug resistance and relapse [60].

#### 2.6.3. Active site prediction using CASTp server.

To identify the active sites/binding sites, the target proteins’ PDB files were evaluated using the Computed Atlas of Surface Topography of Proteins (CASTp) server. CASTp predicts binding site residues based on geometric and topological properties. This analysis provided detailed insights into the accessible binding pockets and interaction sites within the proteins, enabling precise molecular interaction [62].

#### 2.6.4. Molecular docking.

For molecular docking, the selected target proteins were prepared by eliminating water molecules and cofactors to prevent non-specific interactions before docking the ligands. The target proteins were then loaded into PyRx 0.8, where the AutoDock Vina module was used to create PDBQT files for docking analysis. PyRx 0.8 was selected for its computational efficiency, reliability and user-friendly interface, making it one of the most widely used molecular docking platforms. The software allows high-throughput screening of ligands and provides detailed binding affinity scores and interaction profiles.

Thymoquinone, a bioactive compound from *N. sativa* was chosen as a ligand. The canonical SMILES notation for Thymoquinone were retrieved from the PubChem database and converted to PDB format using NovoPro Labs bioscience tools https://www.novoprolabs.com/tools/smiles2pdb). The PDB structure of Thymoquinone was then converted to PDBQT format and underwent energy minimization in PyRx 0.8 to ensure optimal ligand conformation for docking. The same procedure was applied to Busulfan. A grid box was constructed near the binding pockets with coordinates detailed in Table 2 and was subsequently used for docking. Binding interaction visualizations between Thymoquinone, Busulfan and the potential cancer targets were performed using Biovia Discovery Studio. The docking results provided insights into binding affinity, hydrogen bonding patterns, hydrophobic interactions and other non-covalent interactions, highlights the potential of Thymoquinone to modulate the activity of leukemia-associated proteins.

**Table 2:** X, Y, Z coordinates and dimensions of cancer target binding sites.

S. No.	Targets	PDB ID	Centre			Dimensions (Angstroms)		
			X	Y	Z	X	Y	Z
1.	PTEN	1D5R	29.85	91.19	27.52	26.93	29.47	25.96
2.	MMP2	1CK7	51.26	106.64	150.12	87.89	85.66	65.95
3.	TGF-β3	1TGJ	−9.38	33.32	0.71	15.45	21.02	25.0
4.	CD44	1UUH	2.99	−22.20	7.10	25.0	27.93	31.05
5.	AKT	3MV5	7.19	1.73	17.77	38.63	48.74	39.62
6.	EGFR	3W2S	−1.09	6.01	14.62	48.63	43.87	55.81
7.	LYN	3A4O	−5.72	−22.12	−22.38	40.49	46.33	38.38
8.	VEGFR2	3EFL	43.21	36.44	8.08	65.49	55.67	45.26

## 3. Results and discussion

Plants serve as natural industrial units, that producing a wide range of bioactive compounds that many of which are utilized as therapeutics and marketed as natural medicines/drugs. Herbal remedies have long been considered a source of restorative medication due to their association with religious and cultural traditions. Medicinal plants are often preferred over modern allopathic medicines due to their perceived safety, reduced side effects, better biocompatibility and holistic benefits attributed to their natural composition.

The long history of traditional use, provides empirical evidence of their efficacy and safety of medicinal plants. Natural medicine is valued for its holistic benefits, non-toxic, reduced side effects and biocompatibility, derived from complex plant compounds with historical use.

The complex nature of plant-derived compounds allows them to target multiple biological pathways simultaneously, contributing to their broad therapeutic potential. The perceived advantages of natural medicines such as being non-toxic, having fewer side effects and offering better compatibility with the human body further enhance their acceptance and effectiveness. This underscores the significance of plant-based medicines in both traditional and modern healthcare systems.

For thousands of years, the seeds, oils and extracts of *Nigella sativa* were used as an anticancer agent in Unani, Ayurveda and the Chinese systems of medicine, which originated in the Arab, Indo-Bangla and Chinese cultures, respectively [63]. The last two to three decades have seen a significant amount of contemporary scientific research on nigella’s anticancer properties and the latest research indicates that nigella extracts have cytotoxic effects against different cancer cell lines *in vitro*.

### 3.1. Purification of Thymoquinone

**Fig 3:**
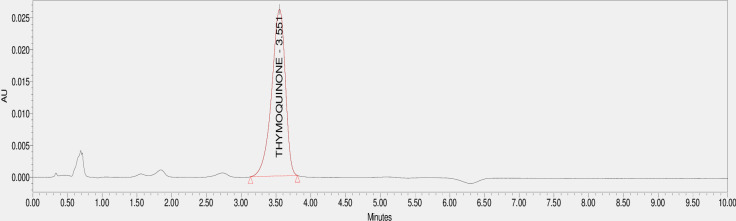
HPLC Chromatogram of Standard Thymoquinone.

**Fig 4:**
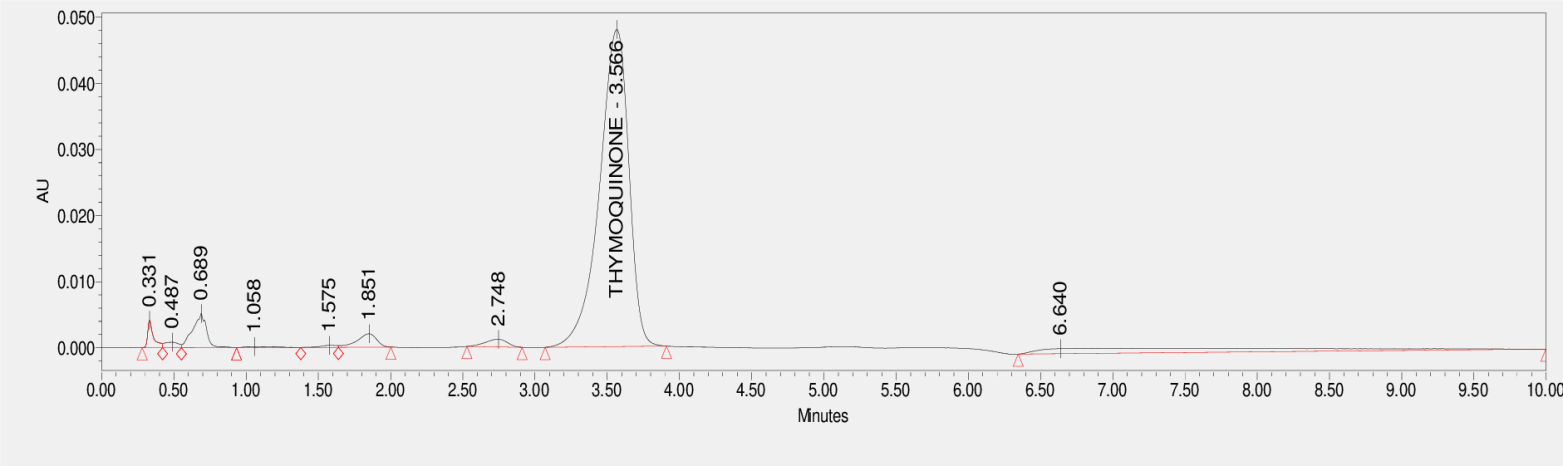
HPLC Chromatogram of Thymoquinone from Seed Oil Extract of Ajmer Nigella-13.

**Fig 5:**
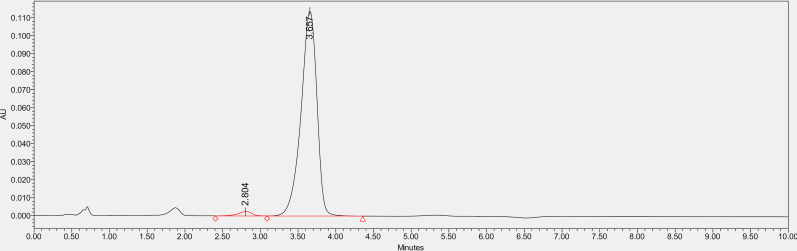
HPLC Chromatogram of Purified Thymoquinone.

The existence of Thymoquinone extracted in methanol from *N. sativa* L., seed was proven with the help of HPLC, which contrasted the extract’s retention time with the standard Thymoquinone ( 3.551 min) (Fig 3). The studied accession Ajmer Nigella 13 reported a peak area similar to the standard with the time of 3.566 minutes (Fig 4). further the total oil extracted via Soxhlet extraction was used for purification of Thymoquinone metabolite via column chromatography and it was confirmed via TLC (Fig 1) and isolated pure Thymoquinone was obtained (Fig 5) at 3.657 minutes retention time. .

### 3.2. In-vitro anticancer activity of Thymoquinone

The cytotoxicity study *(in vitro*) was executed in human myelogenous leukaemia (K562) cell line using different concentrations of purified Thymoquinone at 5 µM ml^−1^, 25 µM ml^−1^, 50 µM ml^−1^, 75 µM ml^−1^, 100 µM ml^−1^ for 24, 48 and 72 hours’ duration (Fig 2). When the isolated metabolite was tested for 24 hours duration at 5, 25 and 50 µM/ml has reported 50%, 63% and 72% cytotoxicity respectively indicated moderately cytotoxic and at 75 µM ml^−1^ and 100 µM ml^−1^ yielded 81% and 84% cytotoxicity respectively has showed severely cytotoxic. Further the same five different concentrations for 48 and 72 hours duration have recorded severe cytotoxic with values of 58, 66%, 73%, 83%, 88% and 75%, 81%, 82%, 85% and 90% respectively. This indicated that the viability of K562 cells by a concentration and time-dependent increase in cell cytotoxicity in accordance with the recommendations of ISO 10993–5:2009 and the metabolite Thymoquinone was found to exhibit strong anticancer activity.

The results showed that isolated Thymoquinone has a significant *in vitro* cytotoxic effect against human myelogenous leukaemia (K562) cell line in a dose or concentration-dependent manner. The earlier studies on the anticancer properties of Thymoquinone have been reported and it was due to its cells cycle arrest and induction of proapoptotic effects in cancer-causing cells. The results are also in line with [64] where the metabolite Thymoquinone inhibited the proliferation of neoplastic keratinocytes in mice up to 50% at non-cytotoxic concentration level. Another study found that Thymoquinone causes apoptosis in human umbilical vein endothelial cells and inhibits VEGF-dependent ERK and Akt activation [65] found that Thymoquinone promotes apoptosis in mammalian umbilical vein cells that are endothelial inhibited VEGF-dependent ERK and the Akt activation and decreased the development of a series of cancer cells from humans (Caco-2, HT-29) by bringing together the process of phosphorylation of mitogen-activated protein kinases (MAPK), JNK and ERK [66]. Our findings reveal that the isolated Thymoquinone effectively inhibited the proliferation of human myelogenous leukemia (K562) cells in dose and time dependent manner, even at lower concentrations demonstrating significant cytotoxic activity.

### 3.3. Changes in cell morphology

**Fig 6:**
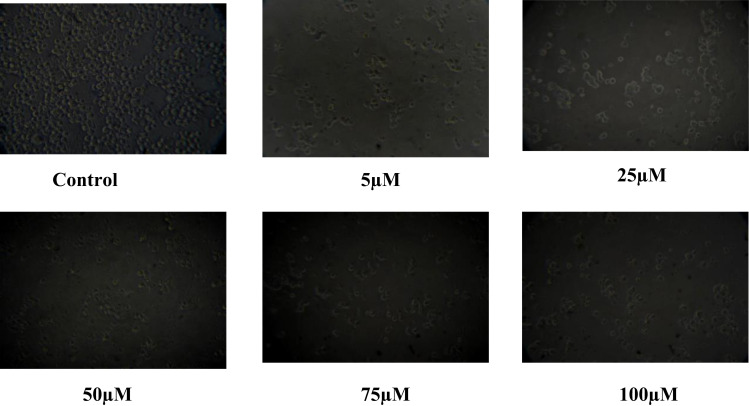
Morphological Changes in K562 Cells Exposed to various Concentrations of Thymoquinone for 24 hours.

**Fig 7:**
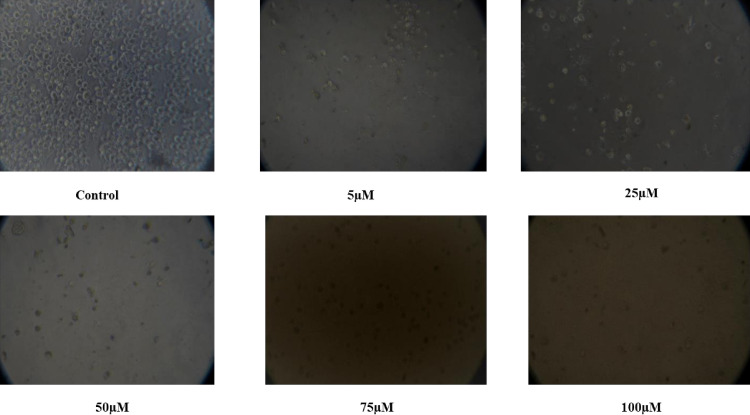
Morphological Changes in K562 Cells Exposed to Various Concentrations of Thymoquinone for 48 hours.

**Fig 8:**
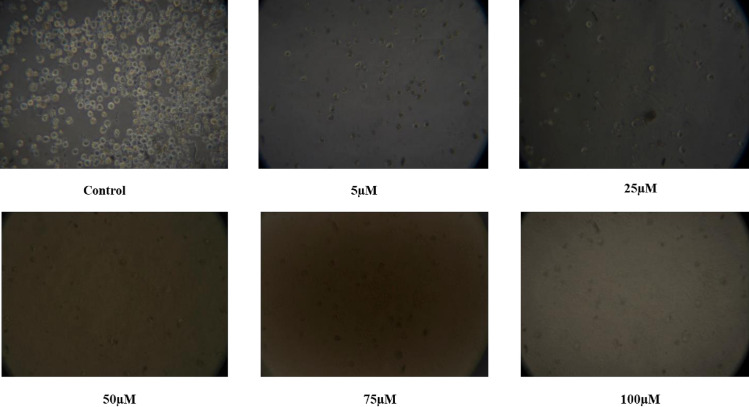
Morphological Changes in K562 Cells Exposed to Various Concentrations of Thymoquinone for 72 hours.

Studying morphological changes in cell lines for anti-cancer assays *in vitro* provides a powerful, cost-effective and insightful approach to understanding drug effects. The morphological changes in cells can be early indicators of the effectiveness of anti-cancer compounds and help to identify specific cellular structures or pathways targeted by anti-cancer drugs. The advanced imaging techniques allow for real-time monitoring of morphological changes, providing dynamic insights into the effects of anti-cancer agents over time. The cell morphology of K562 cells exposed to isolated Thymoquinone metabolite changed in a dose or concentration and time-dependent way. It is evident from Fig 6 that the cells exposed to 5 µM ml^−1^ concentrations exhibited decreased morphology and cell adhesive capacity compared to the control. Further, most cells exposed to higher concentrations of isolated Thymoquinone lost their lost their characteristic shape and appeared smaller in size. The results of our stay are in comparison with a study by [67] wherein the extracts of *N. sativa* seed and *N. sativa* seed oil inhibited cancer cell proliferation and affected the cellular shape of A-549 cells in a concentration-dependent way [Fig 7, 8].

### 3.4. Predication of ADMET properties of Thymoquinone and Busulfan

The Table 3 provides a comparison between Thymoquinone (C_10_H_12_O_2_) and Busulfan (C_6_H_14_O_6_S_2_) across several physiochemical characteristics. Thymoquinone has a lower molecular weight (164.2) with 1 rotatable bond, 2 hydrogen bond acceptors and no hydrogen bond donors, while Busulfan is heavier (246.3) with 7 rotatable bonds, 6 hydrogen bond acceptors and no hydrogen bond donors. Both compounds display high gastrointestinal absorption, but only Thymoquinone is capable of crossing the blood-brain barrier. Neither compound acts as a P-glycoprotein substrate or inhibits CYP enzymes. Thymoquinone shows a skin permeability (log Kp) of −5.74, compared to Busulfan’s −8.17. Importantly, neither compound violates Lipinski, Ghose, Veber and Egan drug-likeness rules, both maintain a bioavailability score of 0.55.

**Table 3:** Predicted ADMET properties of Thymoquinone and Busulfan.

Physiochemical properties	Compound Name	
	Thymoquinone	Busulfan
**Formula**	C10H12O2	C6H14O6S2
**Molecular weight**	164.2	246.3
**Rotatable bonds**	1	7
**H-bond acceptors**	2	6
**H-bond donors**	0	0
**Consensus Log P**	1.85	0.52
**GI absorption**	High	High
**BBB permeant**	Yes	No
**Pgp substrate**	No	No
**CYP enzymes inhibitors**	No	No
**log Kp (cm/s)**	−5.74	−8.17
**Lipinski**	Accepted	Accepted
**Ghose**	Accepted	Accepted
**Veber**	Accepted	Accepted
**Egan**	Accepted	Accepted
**Bioavailability Score**	0.55	0.55

Thymoquinone, with its lower molecular weight, fewer rotatable bonds and ability to cross the blood-brain barrier, shows promising physiochemical characteristics for drug development. Its better skin permeability compared to Busulfan and compliance with drug-likeness rules further suggest that Thymoquinone could be a viable alternative to synthetic drugs like Busulfan, potentially offering benefits in terms of absorption and effectiveness.

ADMET properties in *in-silico* approaches predict how compounds are absorbed, distributed, metabolized and excreted and their toxicity. This helps identify viable drug candidates and reduce risks early in development. By evaluating these properties computationally, researchers can streamline drug development, saving time and resources [68]. Natural compounds can provide broader therapeutic effects and fewer side effects than synthetic drugs, along with traditional use and cultural acceptance [69].

Thymoquinone’s natural origin, combined with its predicted ADMET profile, supports its potential as a safer and more effective alternative to synthetic drugs like Busulfan. Further exploration of its mechanisms and therapeutic potential could lead to the development of novel, plant-derived anticancer alternatives.

### 3.5. Computation approach – Molecular docking

**Fig 9:**
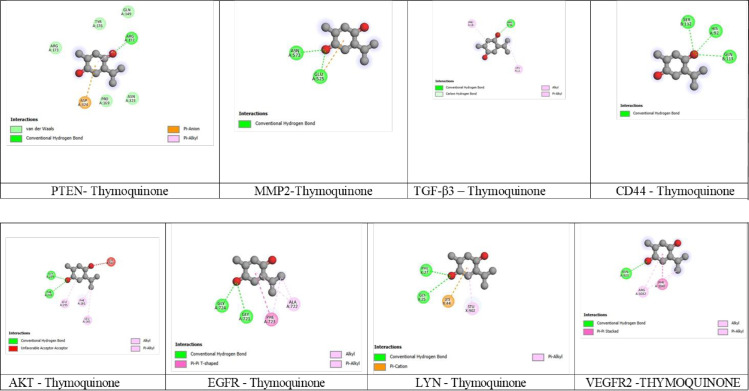
Interaction of Thymoquinone with Different Leukemia Targets.

**Fig 10:**
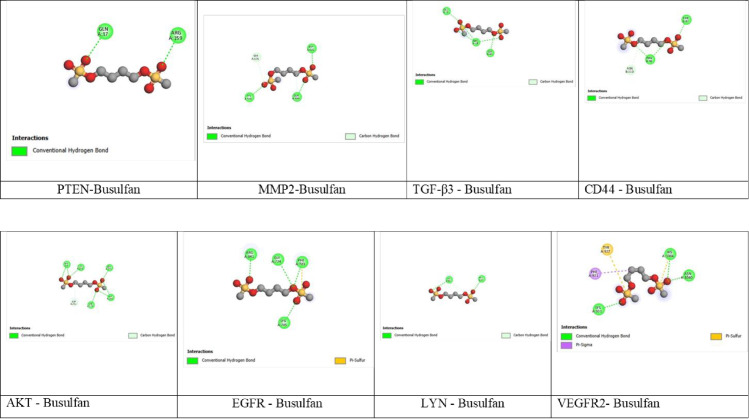
Interaction of Busulfan with Different Leukemia Targets.

Molecular docking revealed that Thymoquinone interacts with eight different cancer targets and demonstrates a lower binding energy compared to the standard, Busulfan. The predicted binding interactions of Thymoquinone and Busulfan are illustrated in Fig 9 & 10. and binding scores in terms of energy values are given in Table 4. The binding energy scores for the interaction between the targets and the Thymoquinone ranged from ^−^6.7 to −4.4 kcal mol^−1^.

**Table 4:** Predicted interaction of Thymoquinone and Busulfan with cancer targets.

S. No.	Target proteins and their PDB ID	Binding energy (kcal/mol)		No. of Hydrogen bond		Interacting active site residues	
		Thymoquinone	Busulfan	Thymoquinone	Busulfan	Thymoquinone	Busulfan
**1.**	PTEN (1D5R)	−5.5	−4.4	1	2	ARG172:A	GLN17:A, ARG159:A
**2.**	MMP2 (1CK7)	−6.7	−5.8	2	3	GLU525:A, ASN573:A	GLN480:A, LYS579:A ASP622:A
**3.**	TGF-β3 (1TGJ)	−4.4.	−4.4	1	5	ARG18:A	GLU12:A, ARG18:A SER45:A
**4.**	CD44 (1UUH)	−4.1	−4.7	3	3	HIS92:A SER112:B GLN113:B	ARG46:B THR47:B
**5.**	AKT 3MV5	−6.0	−5.3	2	7	PHE293:A, GLY294:A	SER7:A, LYS179:A LYS276:A, GLY294:A LEU295:A
**6.**	EGFR (PDB:3W2S)	−5.5	−4.9	2	4	GLY721:A,GLY724:A	PHE723:A, GLY724:A LYS745:A, ARG841:A
**7.**	LYN 3A4O	−5.3	−4.6	2	2	GLY25:X, PHE27:X	PHE27:X, LYX44:X
**8.**	VEGFR2 3EFL	−5.2	−4.2	1	3	ASN923:A	LYS931:A, HIS1004:A ASN1040:A

Among the eight targets, MMP2 (PDB: 1CK7) displayed the lower binding energy of −6.7 kcal mol^−1^ compared to the standard drug, which had a binding value of −5.8 kcal mol^−1^ and formed two hydrogen bonds with GLU525 and ASN573 of the A chain, while Busulfan interacted with GLN480, LYS579 and ASP622 of the A chain. After MMP2, AKT (PDB: 3MV5) exhibited a binding energy score of −6.0 kcal mol^−1^ and formed two hydrogen bonds with PHE293 and GLY732 of A chain (Table 4, Fig 9).

PTEN (PDB:1DR5) and EGFR (PDB: 3W2S) both displayed the same binding energy score of −5.5 kcal mol^−1^ with Thymoquinone and interacted with ARG172 in PTEN (Fig 9) and GLY 721 and GLY724 in EGFR (Fig 9), respectively. While, Busulfan displayed binding energies of −4.4 kcal mol^−1^ for PTEN and −4.9 kcal mol^−1^ for EGFR respectively (Table 4). It formed two hydrogen bonds with GLN17 and ARG159 in PTEN (Fig 10) and interacted with four residues PHE723, GLY724, LYS745 and ARG841 in EGFR (Fig 10).

LYN (PDB: 3A4O) interacted with Thymoquinone exhibited a binding energy score of −5.3 kcal mol^−1^ and formed two hydrogen bonds with GLY 25 and PHE 27 of the X chain while, Busulfan had a binding score of −4.6 kcal mol^−1^ and interacted with PHE 27 and LYX 44 of the X chain (Table 4, Fig 9 and 10).

VEGFR2 (PDB: 3EFL) displayed a binding energy score of −5.2 kcal mol^−1^ with Thymoquinone and interacted with ASN 923 in chain A, while Busulfan displayed −4.2 kcal mol^−1^ and interacted with three residues LYS931, HIS1004 and ASN 1040 in chain A (Table 4, Fig 9 and 10).

TGF-β3 interacted with both Thymoquinone and Busulfan displayed the same binding energy of −4.4 kcal mol^−1^. Thymoquinone formed a hydrogen bond with ARG 18 of chain A and Busulfan interacted with GLU 12, ARG 18 and SER 45 of the chain A (Table 4, Fig 9 and 10).

CD44 (PDB: 1UUH) exhibited a binding value of −4.1 kcal mol^−1^ formed three bonds of hydrogen with HIS92 in chain A and SER112 and GLN133 in chain B, while, Busulfan had a least binding energy score of - 4.7 kcal mol^−1^ and interacted with ARG46 and THR47 of chain B (Table 4, Fig 9 and 10).

The molecular docking analysis revealed that Thymoquinone exhibited a least binding energy affinity compared to the standard drug, Busulfan for most of the selected oncogenic targets, including MMP2, AKT, PTEN and EGFR. The least binding affinity was observed with MMP2 (PDB: 1CK7) at −6.7 kcal/mol, indicating that Thymoquinone strongly interacts with matrix metalloproteinases involved in tumor invasion and metastasis (25 and 29). The interaction of Thymoquinone with AKT (PDB: 3MV5) at −6.0 kcal/mol suggests its potential to modulate the PI3K/AKT signaling pathway, which is crucial for leukemia cell survival and proliferation [70,71].

Significant interaction was also observed with PTEN (PDB: 1D5R) and EGFR (PDB: 3W2S), with same binding affinities of −5.5 kcal/mol. The interaction with PTEN suggests that Thymoquinone may restore tumor-suppressor function and inhibit downstream oncogenic signaling [72], Moderate interaction with VEGFR2 (PDB: 3EFL) (−5.2 kcal/mol) highlights Thymoquinone’s potential anti-cancer activity [73].

The relatively lower binding affinity for TGF-β3 (−4.4 kcal/mol) and CD44 (−4.1 kcal/mol) suggests that Thymoquinone’s anticancer activity is primarily mediated through inhibition of cell proliferation and invasion rather than immune modulation or cell adhesion. The overall docking results indicate that Thymoquinone exhibits a multi-targeted mechanism, effectively inhibiting key pathways involved in various stages/progression of cancer.

Thymoquinone exhibits a multi-targeted anticancer mechanism by inhibiting key pathways involved in cancer progression, including proliferation, survival and metastasis. It disrupts the PI3K/AKT pathway (via AKT and PTEN), limits growth factor signaling (via EGFR and VEGFR2) and reduces metastatic potential (via MMP2 and CD44). This highlights strongly demonstrate its broad-spectrum by targeting multiple hallmarks of cancer.

*In-silico* docking and ADMET predictions of Thymoquinone derivative Thy09 highlighted its potential against ovarian cancer. The analysis revealed favourable binding and pharmacokinetic profiles, suggesting Thy09 as a promising candidate for effective anti-cancer treatment [74,75] investigated Thymoquinone’s role in combating cancer and metastasis by regulating key oncogenic signalling pathways. It demonstrated potential as an effective therapeutic agent by disrupting critical cancer-related signalling mechanisms.

Thymoquinone’s ability to inhibit K562 cell proliferation, invasion and survival through multi-targeted action highlights its potential as a promising anti-leukemic compound. The strong correlation between the molecular docking and cytotoxicity data suggests that its primary mechanism of action involves inhibition of the PI3K/AKT pathway and extracellular matrix remodelling (via AKT, PTEN and MMP2), with secondary contributions from angiogenesis (via VEGFR2) and immune modulation (via TGF-β3 and CD44).

These findings position Thymoquinone as a potent candidate for future preclinical and clinical evaluation in leukemia treatment, offering a natural, targeted alternative with broad-spectrum activity against key oncogenic genes.

## 4. Conclusion

Plant-derived anticancer compounds are gaining recognition for their safety, accessibility and multi-targeting capabilities, making them promising candidates for leukemia treatment. In this context, *Nigella sativa* L. seeds have been widely explored and Thymoquinone identified as a key bioactive compound. Human myelogenous leukemia (K562) remains a significant therapeutic challenge due to drug resistance and the need for more effective treatment options. The alarming rise in leukemia cases necessitates the exploration of alternative, targeted therapies with improved efficacy and no side effects.

In this study, the quantification of Thymoquinone from Ajmer Nigella 13 accession was followed by in vitro cytotoxicity revealed a significant dose- and time-dependent inhibition of K562 cell proliferation and survival. The *In Silico* molecular docking analysis confirmed that Thymoquinone strongly interacted with key cancer targets like MMP2, AKT, PTEN and VEGFR2 with least binding energy than standard drug, Busulfan. This indicates that Thymoquinone may inhibit PI3K/AKT pathway, extracellular matrix remodelling and angiogenesis, reducing cancer cell growth and spread.

The combined *in vitro* and *in silico* findings highlight Thymoquinone’s multi-targeted mechanism of action, positioning it as a potent natural alternative for leukemia treatment. Further preclinical and clinical studies are needed to confirm these findings and investigate the therapeutic potential of Thymoquinone in leukemia treatment.
